# Activation of RARα Receptor Attenuates Neuroinflammation After SAH *via* Promoting M1-to-M2 Phenotypic Polarization of Microglia and Regulating Mafb/Msr1/PI3K-Akt/NF-κB Pathway

**DOI:** 10.3389/fimmu.2022.839796

**Published:** 2022-02-14

**Authors:** Yang Tian, Binbing Liu, Yuchen Li, Yongzhi Zhang, Jiang Shao, Pei Wu, Chao Xu, Guangduo Chen, Huaizhang Shi

**Affiliations:** ^1^ Department of Neurosurgery, First Affiliated Hospital of Harbin Medical University, Harbin, China; ^2^ Department of Cardiology, Fujian Medical University Union Hospital, Fuzhou, China

**Keywords:** subarachnoid hemorrhage, retinoic acid receptor α, tamibarotene, Msr1, neuroinflammation

## Abstract

**Background and Purpose:**

Subarachnoid hemorrhage (SAH) is a life-threatening subtype of stroke with high rates of mortality. In the early stages of SAH, neuroinflammation is one of the important mechanisms leading to brain injury after SAH. In various central nervous system diseases, activation of RARα receptor has been proven to demonstrate neuroprotective effects. This study aimed to investigate the anti-inflammatory effects of RARα receptor activation after SAH.

**Methods:**

Internal carotid artery puncture method used to established SAH model in Sprague-Dawley rats. The RARα specific agonist Am80 was injected intraperitoneally 1 hour after SAH. AGN196996 (specific RARα inhibitor), Msr1 siRNA and LY294002 (PI3K-Akt inhibitor) were administered *via* the lateral ventricle before SAH. Evaluation SAH grade, neurological function score, blood-brain barrier permeability. BV2 cells and SH-SY5Y cells were co-cultured and stimulated by oxyhemoglobin to establish an *in vitro* model of SAH. RT-PCR, Western blotting, and immunofluorescence staining were used to investigate pathway-related proteins, microglia activation and inflammatory response. Results: The expression of RARα, Mafb, and Msr1 increased in rat brain tissue after SAH. Activation of the RARα receptor with Am80 improved neurological deficits and attenuated brain edema, blood brain barrier permeability. Am80 increased the expression of Mafb and Msr1, and reduced neuroinflammation by enhancing the phosphorylation of Akt and by inhibiting the phosphorylation of NF-κB. AGN196996, Msr1 siRNA, and LY294002 reversed the therapeutic effects of Am80 by reducing the expression of Msr1 and the phosphorylation of Akt. *In vitro* model of SAH, Am80 promoted M1-to-M2 phenotypic polarization in microglia and suppressed the nuclear transcription of NF-κB.

**Conclusion:**

Activation of the RARα receptor attenuated neuroinflammation by promoting M1-to-M2 phenotypic polarization in microglia and regulating the Mafb/Msr1/PI3K-Akt/NF-κB pathway. RARα might serve as a potential target for SAH therapy.

## Introduction

Subarachnoid hemorrhage (SAH) is a fatal form of cerebrovascular disease that accounts for 5% - 10% of all stroke types and represents a serious public health problem (1). Early brain injury (EBI) is an important factor underlying the high rates of mortality and disability in patients after SAH. Accumulating evidence indicates that EBI is a complex pathological process that includes many pathological mechanisms, including neuroinflammation, oxidative stress, apoptosis, dysfunction of the blood-brain barrier (BBB), and ferroptosis (2–4). Activated microglia-mediated neuroinflammation has also been considered as a potential participant in the pathogenesis of EBI (5). Within 72 hours of SAH, activated microglia release many inflammatory factors, amplify the inflammatory response, and aggravate nerve function damage. On the other hand, activated microglia are polarized into two different states, M1 (pro-inflammatory) and M2 (anti-inflammatory). M1 microglia aggravates brain damage by releasing pro-inflammatory cytokines, including *IL-1β*, *TNF-α*, and *IL-6*. In contrast, M2 microglia provide neuroprotective effects by releasing anti-inflammatory factors (6, 7). Furthermore, activated microglia can transform between M1 and M2 phenotypes depending on the microenvironment and the intertwined intracellular signals (6). Therefore, inhibiting the excessive activation of microglia and promoting the polarization of activated microglia to anti-inflammatory phenotypes might be beneficial in the improvement of neurological outcomes after SAH.

Retinoic acid receptors (RARs) belong to the nuclear receptor superfamily and are involved in the regulation of cell differentiation, proliferation, embryonic development, metabolism, and other life activities (8). RARs are encoded by three distinct genes (α, β, and γ) and are known to be expressed in the adult central nervous system (CNS) (9, 10). Previous studies have demonstrated that when activated, RARs can protect neurons from inflammation-associated injury (11, 12) and represent a potential therapeutic target for Alzheimer’s disease (AD) (13). As a member of the nuclear receptor superfamily, RARα exhibits a variety of biological effects by binding to corresponding ligands, thus regulating the transcription of target genes (14). Tamibarotene (Am80), a selective agonist of RARα, has been shown to exert significant inhibitory effects on neuroinflammation after ischemic stroke and intracranial hemorrhage (ICH) (14–17), thus suggesting the suitability of targeting RARα as a form of stroke therapy. However, the role of Am80 in anti-neuroinflammation has not been reported for EBI after SAH.

Macrophage scavenger receptor 1 (Msr1), as a heterogeneous molecule on the surface of phagocytes, was the first scavenger receptor to be characterized (18). In the central nervous system, Msr1 is mainly expressed in microglia. Recent evidence has shown that Msr1 participates in many pathophysiological events, including inflammation, atherosclerosis, and the recognition of viruses (19, 20). Msr1 may also activate different signaling pathways such as the PI3K-Akt and nuclear factor kappa B (NF-κB) pathways by binding with different ligands (18). V-maf musculoaponeurotic fibrosarcoma oncogene homolog B (Mafb), as an upstream regulatory transcription factor of Msr1, is known to enhance the internalization of damage-associated molecular patterns (DAMPs) by mediating the expression of Msr1 to reduce inflammatory damage (21). Therefore, it is possible that Mafb and Msr1 may also exert protective effects in SAH.

In the present study, we hypothesized that activation RARα receptor reduced neuroinflammation after SAH by promoting the M1-to-M2 phenotypic polarization of microglia and by regulating the Mafb/Msr1/PI3K-Akt/NF-κB pathway.

## Materials and Methods

### SAH Animal Model

Sprague-Dawley rats (male, weight 270–320 g) were acquired from the Animal Center of the First Affiliated Hospital of Harbin Medical University and raised in a room with appropriate humidity and a controlled temperature of 25-28°C. All rats were allowed to eat and drink freely prior to modeling. Intravascular perforation was performed as previously described (22). All animal experiments were carried out in accordance with the guidelines of the National Institutes of Health (NIH) and were approved by the Institutional Animal Care and Use Committee of the First Affiliated Hospital of Harbin Medical University (NO:2020047).

### SAH Grading

Two researchers independently graded the severity of SAH, as described previously (23). In brief, SD rats were sacrificed after the induction of SAH. Next, wo measured the volume of blood clots in different areas of the basal surface of the brain, as follows: no blood clots = 0; a small number of blood clots = 1; a moderate number of blood clots discernible within the arteries = 2 points, and blood clots covering all of the arteries = 3 points. Rats with a SAH grading score < 7 were excluded from subsequent experiments.

### Drug Administration

Am80 (Tocris, Ellisville, USA) was dissolved in carboxymethyl cellulose solution and administered by intraperitoneal (i.p.) injection 1 h after SAH. AGN196996 (Allergan Inc, USA) was diluted in Dimethyl sulfoxide (DMSO) and administered by intracerebroventricular (i.c.v.) injection 1 h prior to SAH. 500 pmol of Msr1 siRNA and scramble RNA (Thermo Fisher Scientific, Waltham, USA), in a total volume of 5 μL was injected (i.c.v.) 48 h prior to SAH. LY294002 (Selleck Chemicals, Houston, USA) was injected (i.c.v.) 2 h prior to SAH. The dose and time points of administration were based on previous research (24, 25).

### *In Vivo* Experimental Protocols

#### Experiment 1

Western blotting was performed in the Sham and SAH models at 3, 6, 12, 24, and 72 hours after SAH (n=6 per group) to determine the endogenous expression of RARα, Mafb and Msr1 in brain tissue. In addition, immunofluorescence staining used to indicate the co-localization of RARα, Mafb and Msr1 with the microglia at 24 hours in the Sham and SAH models after surgery(n=3 per group).

#### Experiment 2

To evaluate the neurological outcome, 30 rats were randomly assigned into 5 groups (n=6 per group): Sham, SAH+vehicle (carboxymethyl cellulose solution), SAH+Am80 (1 mg/kg), SAH+Am80 (5 mg/kg), and SAH+Am80 (10 mg/kg). Compare the neurological scores of each group when tested 24 h after surgery, we chose 5 mg/kg dose of Am80 utilize in subsequent experiments.

#### Experiment 3

To assess neuronal injury and the activation of microglia after SAH, 27 rats (n=9 per group) were randomly assigned into 3 groups: Sham, SAH+vehicle, and SAH+Am80 (5mg/kg). Neuronal damage was investigated by TUNEL staining 24 h after SAH, and Nissl staining 14 days after SAH. In addition, western blotting, and immunofluorescence (Iba-1) were carried out at 24 h after SAH and used to quantify the number of activated microglia in each group. The methods used to analyze the morphology of microglia were reported previously (26).

#### Experiment 4

To investigate the specific role of RARα, 30 rats (n=6 per group) were randomly divided into Sham, SAH+vehicle, SAH+Am80, SAH+AGN196996+Am80, and SAH+DMSO+Am80 groups for western blotting. An additional 15 rats (n=3 per group) were used for immunofluorescence (*TNF-α*).

#### Experiment 5

To investigate underlying molecular mechanisms, 30 rats (n=6 per group) were randomly divided into 5 groups: SAH, SAH+scramble small interfering RNA (siRNA)+Am80, SAH+MSR1 siRNA+Am80, SAH+DMSO+Am80, and SAH+LY294002+Am80. Another 15 rats (n=3 per group) were used for immunofluorescence analyses (*TNF-α*).

### The Assessment of Neurological Outcomes

Neurological outcomes were assessed 24 h after SAH by modified Garcia scores and the beam balance test. The modified Garcia score consists of six aspects, including autonomous activity, autonomous movement of the four limbs, forepaw outstretching, whisker proprioception, body proprioception, and climbing. The score for each part ranges from 0-3 points, and the score used for evaluation is the sum of the scores for each part. Beam balance tests were used to evaluate the ability of rats to balance. In brief, the rats were allowed to walk on a narrow wooden beam for 1 minute; the score ranged from 0 to 4 according to walking distance and the number of falls or slips. As previously described (4), the average scores of three consecutive trials was calculated for analysis, and blindly evaluated by two independent investigators.

### Evaluation of BBB Permeability and Brain Water Content

The permeability of the BBB was evaluated at 24h after SAH, as reported previously (22). In brief, Evan’s blue (2%, 5 mL/kg, Sigma, USA) was injected into the left ventricle 1 h prior to sacrifice. Brain tissues were weighed after perfusion with normal saline, homogenized in 50% trichloroacetic acid (TCA), and then centrifuged. The supernatant was then collected, mixed with ethanol and TCA, and then maintained overnight at 4°C. Evan’s blue concentration determined by absorbance at 630 nm represented BBB permeability.

To evaluate water content in the brain. Rats were sacrificed 24 h after surgery and the brains were divided into four parts: the left cerebral hemispheres, the right cerebral hemispheres, the brain stem, and the cerebellum. Each brain segment was weighed to provide a wet weight. Then, each segment was placed in an oven for 24 h at 100°C to determine the dry weight. The formula used to calculate brain water content was as follows: [(wet weight-dry weight)/wet weight]×100%.

### Nissl Staining

At set time points after SAH, The rats were sacrificed and perfused with cold phosphate buffer solution (PBS) and 4% paraformaldehyde (PFA). Then, fixed the brain in PFA solution at 4°C for 24 h. After embedding the tissue in paraffin, cut into 5um slices for further use. Nissl staining (14 days after SAH) was performed using 5% cresyl blue and observed by light microscopy for neuronal morphology and cell count (24, 27).

### *In Vitro* SAH Model

SH-SY5Y cells (Shanghai, China) were cultured in Dulbecco’s modified Eagle’s medium (DMEM) (Gibco, NY, USA) containing Ham’s F-12 K Nutrient Mixture with 10% fetal bovine serum (HyClone, PA, USA) and 1% penicillin-streptomycin (Gibco), as reported previously (28). BV-2 microglia cells provided generously by Dr. Mou (Harbin Medicine University, HMU, China), were cultured in DMEM containing 10% FBS and 1% penicillin-streptomycin. BV-2 cells and SY5Y were seeded into 6-well plates (Coring Transwell, USA) and incubated at 37°C and 5% CO2 in a humid environment for the follow-up experiment.

Co-cultured cells were assigned into three groups randomly as previously reported (13, 29): control group (incubated with complete medium); Oxyhemoglobin (Hb; Sigma-Aldrich, MO, USA) +Vehicle group (10 μM Hb and 1% DMSO in complete medium); Hb+Am80 group (incubated with Am80 5 μM after 1 hour of Hb induction).

### RT-PCR

Extraction of total RNA from basal brain tissue by Trizol reagent (Takara, Kyoto, Japan). The sample is transcribed and amplified with the kit (cDNA Synthesis Kit, Takara, Kyoto, Japan; SYBR Premix Ex Taq kit Takara, Kyoto, Japan). All procedures were performed in accordance with the manufacturer’s instructions. The primer sequences used for amplification of *IL-6*, *IL-1β*, *TNF-α* and GAPDH have been previously reported (30, 31). All reactions were run in triplicate and quantification of the qRT-PCR results was performed by the 2−^△△^CT method. The primer sequences are shown in **Supplementary Table 1**.

### Flow Cytometry

Flow cytometry and a specific assay kit (Solarbio, China) were used to assess cell death, as reported previously (4). Data were analyzed by NovoCyte Express (ACEA, Biosciences, USA). Dead cell counts were calculated with the following formula: [Annexin V+/PI+ cells/total cells]×100%.

### Cell Viability Assay

The Cell Counting Kit-8 (CCK-8) (Dojindo, Shanghai, China) was used to assessed the viability of SH-SY5Y cells. In brief, a total of 1000 cells in complete medium were cultured in a 96-well plate. Then, CCK-8 reagent (10 μL) was mixed with 90 μL of DMEM to generate a working solution. 100μL of working solution per hole, incubated for 1.5 h. Cell viability was detected 24 h after Hb induction. There were three replicate wells in each group and repeated three times.

### Western Blotting

Western blotting was performed as described previously (4, 30). Total or nuclear proteins from cells were extracted with RIPA lysis solution (Beyotime, China) or nucleoprotein extraction kit (Wanleibio, China), the protocols were performed in accordance with the manufacturer’s instructions. The protein sample loaded in an equal amount, and transferred to the polyvinylidene fluoride (PVDF) membrane after separation by SDS-PAGE electrophoresis. The membrane was incubated overnight at 4°C with the following primary antibodies: anti-RARα (1: 500, bs-0251R, Bioss), anti-Mafb (1:200, HPA005653, Sigma), anti-Msr1 (1:1000, ab123946, Abcam), anti-Iba-1 (1:2000, ab178846, Abcam), anti-phospho-Akt (1:1000, #9271, Cell Signaling Tech), anti-Akt (1:1000, #9272, Cell Signaling Tech), anti-p-NF-kB p65 (1:1000, #3033, Cell Signaling Tech), anti-NF-κB-p6 5 (1:1000, #8242, Cell Signaling Tech), anti-Arg-1 (1:100, ab96183, Abcam), anti-iNOS (1:100, ab178945, Abcam), anti-Bcl-xl (1:2000, ab32370, Abcam), anti-Bcl-2 (1:500, WL01556, Wanleibio), anti-Bax (1:2000, ab32503, Abcam), anti-cleaved caspase-3 (1:500, ab214430, Abcam), anti-IKBα (1:1000, ab32518, Abcam), anti-p-IKB-α (1:5000, ab133462, Abcam), anti-*IL-6* (1:1000, ab259341, Abcam), anti-*IL-1β* (1:1000, ab254360, Abcam), anti-*TNFα* (1:1000, ab255275, Abcam), anti-β-actin (1:2000, ab8226, Abcam) and anti-Histone 3 (1:2000, ab176842, Abcam). The membranes were incubated in appropriate secondary antibodies (2 h, 37°C). Membranes detected with an enhanced chemiluminescence (ECL) reagent kit (NCM biotech, China) after washed three times. Images were analyzed by Image J software (Image J 1.4, NIH, USA).

### Immunofluorescence and TUNEL Staining

Rats were transcardially perfused with ice-cold PBS and 4% PFA 24 h after SAH. Brain samples were then fixed in PFA for 24 h, followed by dehydration in 30% sucrose for 3 days. Next, the brains were treated with OCT embedding agent and frozen at -80°C. A cryostat was then used to cut the brain tissue into 7 μm sections for double immunofluorescence and TUNEL staining. The cells were seeded in a 6-well transwell plate and treated with oxyhemoglobin or Am80 for co-cultivation. Subsequently, cells were fixed with 4% PFA, permeabilized with 0.2% Triton-100 for 15 minutes, and then washed three times with PBS.

For immunofluorescence staining, the slides were blocked with 5% BSA for 1 hour and incubated overnight at 4°C with the following primary antibodies: anti-RARα (1:50, bs-0251R, Bioss), anti-MAFB (1:200, HPA005653, Sigma), anti-MSR1 (1:200, ab123946, Abcam), anti-NeuN (1:2000, ab177487, Abcam), anti-Iba-1 (1:1000, ab5076, Abcam; 1:1000, ab178846, Abcam; 1:1000, ab283319, Abcam), anti-Arg-1 (1:200, ab96183, Abcam), and anti-iNOS (1:200, ab178945, Abcam). After washing with PBS, the sections were incubated with an appropriate secondary antibody for 2 h at room temperature. TUNEL staining was performed to detect apoptotic cell death with Apoptosis Detection Kit (Roche, USA). Neuronal apoptosis was then evaluated by counting the number of TUNEL-positive neurons in the cortex. Six fields per brain were randomly selected, observed, and imaged on a fluorescence microscope (Lecia Microsystems, Germany).

### Statistical Analysis

All data are presented as mean ± standard deviation (SD) or median with interquartile range. Comparisons between two groups were performed using the student’s t-test One-way analysis of variance (ANOVA) was performed if comparisons involved more than two groups. The Mann Whitney U test or Kruskal-Wallis test, followed by the Dunn *post hoc* test, were used to test variables that were not normally distributed. Statistical analyses were performed using GraphPad Prism software 8.0 (GraphPad Software, US). *P* < 0.05 was defined as statistically significant.

## Results

### SAH Model Mortality and Grade Score

**Figure 1:**
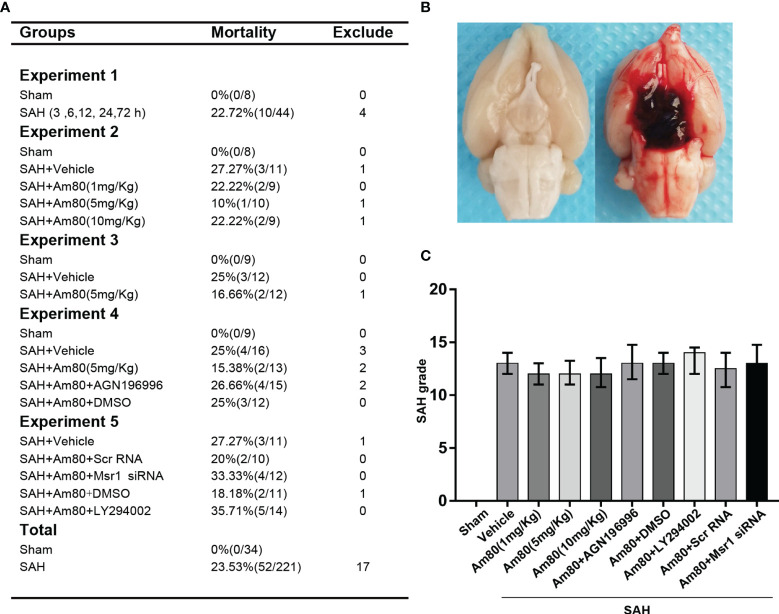
Images of mortality, brain tissues and SAH grading in rats. **(A)** Animal usage and mortality. **(B)** Representative Image showing that blood clot in the subarachnoid space of the rat at 24 h after SAH. **(C)** SAH grading scores in each group. Error bars are represented as medians with interquartile range and analyzed by the Kruskal-Wallis test followed by Dunn’s *post hoc* test.

34 rats were assigned to the Sham group, 221 rats were used to establish the SAH model, and 17 rats were ruled out (SAH grade score <7). The overall mortality rate of SAH modelling was 23.53% (52/221), (**Figure 1A**). Schematic diagram of successful modeling of subarachnoid hemorrhage (**Figure 1B**). There was no significant difference in the SAH grade score of all the SAH groups (**Figure 1C**).

### Temporal Expression of RARα, Mafb, and Msr1

**Figure 2:**
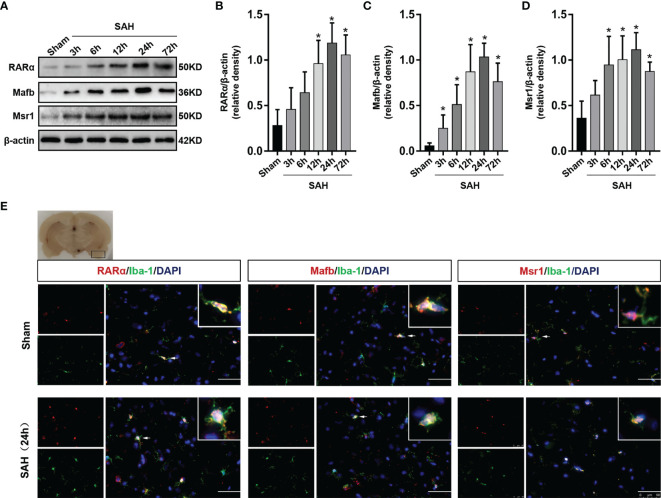
Expression changes of RARα, Mafb and Msr1. **(A–D)** Representative Western blot bands of time course and quantitative analyses for RARα, Mafb, and Msr1. n = 6 per group. *P< 0.05 *vs* Sham group. **(E)** Representative images of double immunofluorescence staining for RARα, Mafb and Msr1 with microglia at 24 h after SAH. n = 3 per group. Scale bar = 50 μm.

Western blotting to detect the expression of RARα, Mafb and Msr1 (**Figure 2A**). The expression of RARα, Mafb and Msr1 began to increase at 3 h and reached a peak at 24 hours after SAH (*P* < 0.05, **Figures 2B–D**). Double immunofluorescence staining further confirmed that RARα, Mafb, and Msr1, co-localized with microglia in the cerebral cortex (**Figure 2E**).

### Activation of RARα Reduced Neuro Injury and Improve the Neurological Score After SAH

**Figure 3:**
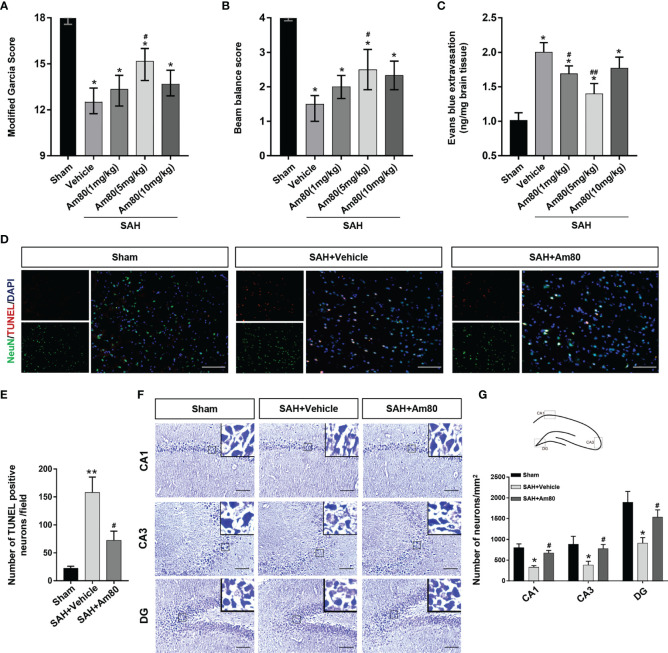
Am80 attenuated neurological deficits, neuronal apoptosis, and BBB permeability at 24 h after SAH. Moreover, Am80 reduced hippocampus injury 14 days after SAH. **(A, B)** The modified Garcia and beam balance scores of each group. n = 6 per group. **(C)** Quantification of Evans blue extravasation at 24 h after SAH. **(D)** Representative images of TUNEL staining (red) in neurons (NeuN, green). **(E)** Quantitative analyses of TUNEL-positive cells. Scale bar = 100 μm. n=3 per group. **(F)** Representative images of Nissl staining in the CA1, CA3, and DG regions. **(G)** Upper middle indicates the hippocampus division and location of staining. Quantitative analyses of Nissl staining. n=3 per group. Scale bar = 100 μm. *P < 0.05 *vs* sham group; **P < 0.01 *vs* sham group; ^#^ *P* < 0.05 *vs* SAH+vehicle group; ^##^ *P* < 0.05 *vs* SAH+vehicle group.

The neurological function of rats was scored by modified Garcia score and beam balance tests at 24 hours after SAH. The neurobehavioral scores were significantly reduced after SAH surgery when compared with the Sham group (*P* < 0.05, **Figures 3A, B**). Am80 administration reversed the decline in neurological score (the modified Garcia score and beam balance tests) (*P* < 0.05, **Figures 3A, B**). After SAH surgery, the extravasation of Evan’s blue was significantly increased, whereas, Am80 treatment reversed the extravasation. (*P* < 0.05, **Figure 3C**). The brain water content weighing result displayed the extent of brain edema after SAH surgery on both hemispheres was significantly higher than Sham group 24h after SAH (*P* < 0.05, **Supplementary Figure 1A**). Treating with Am80 administration, the brain edema of the both hemispheres was significantly alleviated after SAH (*P* < 0.05, **Supplementary Figure 1A**). These results suggested that optimal therapeutic dosage of Am80 was 5 mg/kg after SAH in rats.

We used TUNEL staining to evaluate neuronal apoptosis. The results showed that 24 hours after SAH induction, the number of apoptotic neurons increased significantly in the SAH+Vehicle group (*P* < 0.05, **Figures 3D, E**). In contrast, the administration of Am80 inhibited neuronal apoptosis (*P* < 0.01, **Figures 3D, E**). Nissl staining showed that 14 days after SAH, the loss of neurons in the Cornu Ammonis area (CA) 1, CA3, and the dentate gyrus (DG), had increased and the morphology of the neurons exhibited shrinkage (*P* < 0.05, **Figures 3F, G**). Am80 significantly reduced the loss of neurons in these parts of the hippocampus (*P* < 0.05, **Figures 3F, G**).

### Activation of RARα Inhibited Neuronal Death *In Vitro* Model of SAH

**Figure 4:**
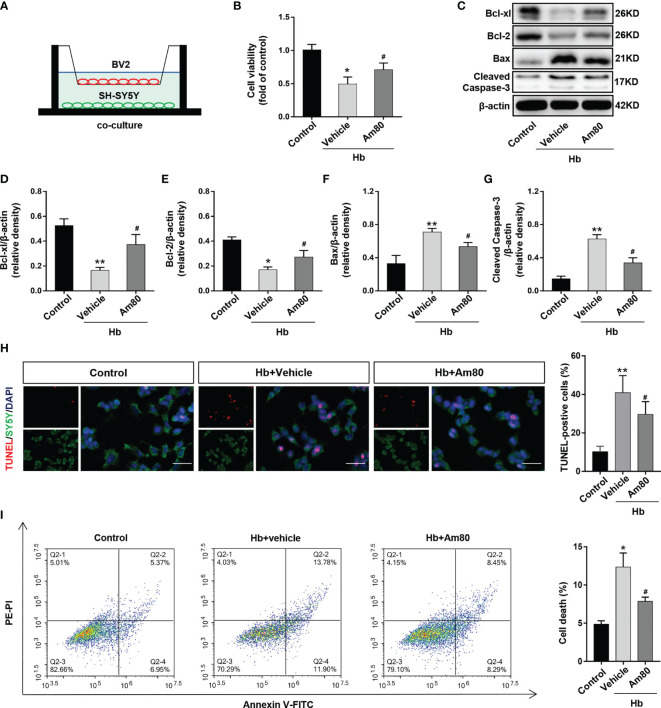
Am80 attenuated neuronal apoptosis *in vitro* SAH model. **(A)** Illustration of the co-culture system. **(B)** Quantitative analyses of cell viability as detected by CCK8 assays. n=3 per group. **(C–G)** Western blotting and quantitative analyses of the expression of Bcl-xl, Bcl-2, Bax, and cleaved-caspase 3 of SH-SY5Y after co-cultivation. n=3 per group. **(H)** Immunofluorescence staining of TUNEL (red) and SH-SY5Y (green) in an *in vitro* co-culture system. Scale bar=50 μm. n=3 per group. **(I)** Representative flow cytometry images of SH-SY5Y cells, the necroptosis is represented by the ratio of PI +/annexin V+. n=3 per group. *P < 0.05 *vs* control group; **P < 0.01 *vs* control group; ^#^ *P* < 0.05 *vs* Hb+vehicle group.

BV2 cells and SH-SY5Y cells were co-cultured and induced by oxyhemoglobin to establish an *in vitro* model of SAH (**Figure 4A**). The results of CCK8 cell viability measurement indicated that the cell viability of the Hb+Vehicle group decreased compared to control group (*P <*0.05, **Figure 4B**). Conversely, Am80 administration reversed this change (*P <*0.05, **Figure 4B**).

Compared with the control group, the expression of Bcl-xl and Bcl-2 decreased after Hb induction, while the expression of Bax and Cleaved-Caspase 3 increased (*P <*0.05, **Figure 4C–G**). AM80 administration reversed the changes in the expression of apoptosis-related proteins induced by Hb (*P <*0.05, **Figure 4C–G**). The TUNEL staining results were consistent with the protein results. AM80 administration reduced the number of apoptotic neurons induced by Hb (*P <*0.05, **Figure 4H**). The results of flow cytometry on apoptotic neurons are consistent with the above results (*P <*0.05, **Figure 4I**).

### Activation of RARα Improved the Abnormal Morphology and Function of Microglia After SAH

**Figure 5:**
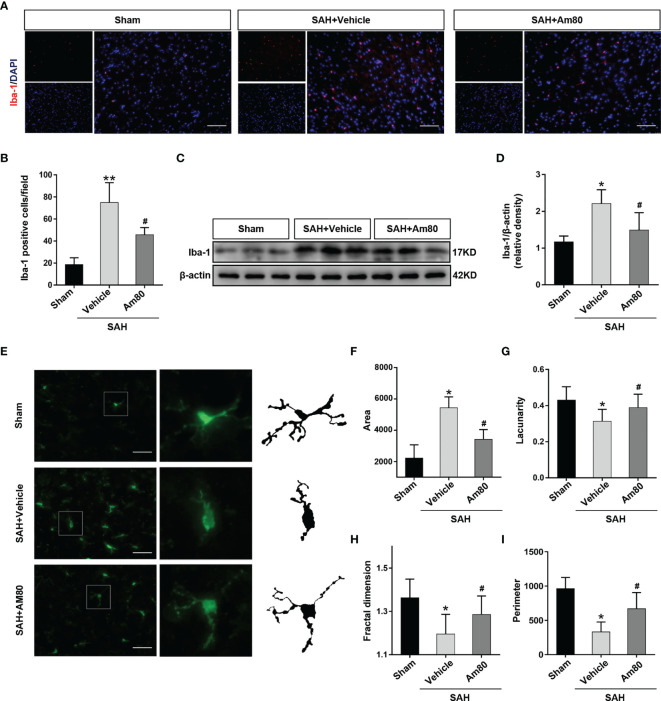
Am80 attenuated the excessive activation of microglia after SAH. **(A, B)** Immunofluorescence staining and quantitative analysis of activated microglia (Iba-1). Scale bar = 100 μm. n = 3 per group. **(C, D)** Western blotting and quantitative analyses of Iba-1 expression. n=6 per group. **(E–I)** Representative images of activated-microglia (green) used for the measurement of morphological parameters and quantification analysis, including area, lacunarity, fractal dimension and perimeter in the basal cortex. Scale bar = 50 μm. n=6 per group. *P < 0.05 *vs* sham group; **P < 0.01 *vs* sham group; ^#^ *P* < 0.05 *vs* SAH+vehicle group.

The results of Iba-1 immunofluorescence suggested that compared with the Sham group, the number of activated microglia increased after SAH (*P <*0.01, **Figures 5A, B**). Am80 treatment significantly reduced the number of Iba-1 positive microglia in the cerebral cortex (*P <*0.01, **Figures 5A, B**). The western blot results of Iba-1 are consistent with the above results (*P <*0.05, **Figures 5C, D**). The area of activated-microglia was increased after SAH, but the lacunarity, fractal dimension, and perimeter were reduced than the Sham group (*P <*0.05, **Figures 5E–I**); Am80 treatment reversed this alteration (*P <*0.05, **Figures 5E–I**).

### Activation of RARα Promoted M1-To-M2 Phenotypic Polarization of Microglia *In Vitro* Model of SAH

**Figure 6:**
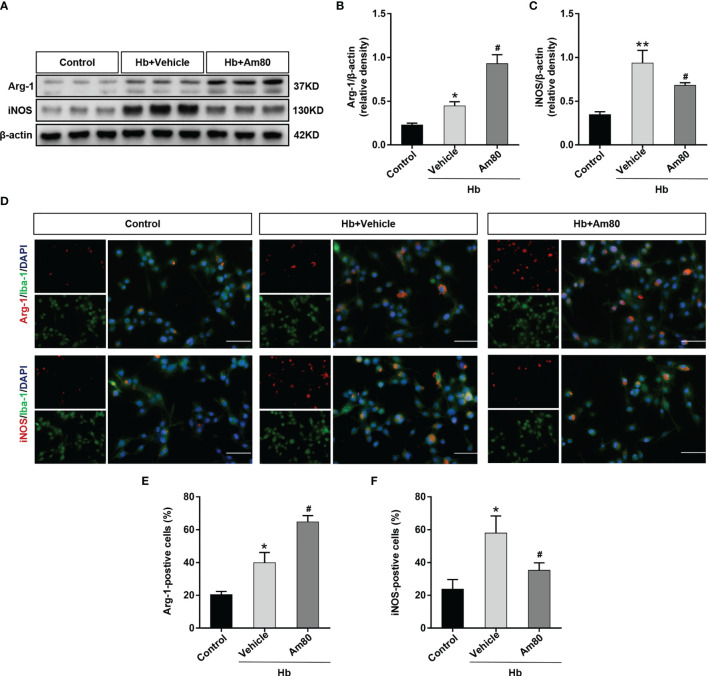
Am80 promoted M1-to-M2 phenotypic polarization of microglia in an *in vitro* model of SAH. **(A–C)** Western blotting and quantitative analyses Arg-1 and iNOS expression of microglia in an *in vitro* co-culture system. n=3 per group. **(D–F)** Immunofluorescence staining of Arg-1/iNOS and microglia in an *in vitro* co-culture system and quantitative analyses of Arg-1/iNOS positive cells. Scale bar=50 μm. n = 3 per group. *P < 0.05 *vs* control group; **P < 0.01 *vs* control group; ^#^ *P* < 0.05 *vs* Hb+vehicle group.

In the *in vitro* SAH model, the expression of Arg-1 and iNOS in BV2 cells increased after Hb induction compared with the control group (*P* < 0.05, **Figures 6A–C**). After Am80 treatment, the expression of Arg-1 further increased, on the contrary, the expression of iNOS decreased (*P* < 0.05, **Figures 6A–C**). Immunofluorescence staining further confirmed that the number of microglia that were positive for Arg-1 and iNOS increased significantly after induced with Hb (P < 0.05, **Figures 6D–F**). After Am80 treatment, the number of M2 type microglia (Arg-1-positive) increased, conversely, the number of M1 type microglia (iNOS positive) decreased (P < 0.05, **Figures 6D–F**).

### Inhibition of RARα Reversed the Anti-Neuroinflammation Effects of Am80 After SAH

**Figure 7:**
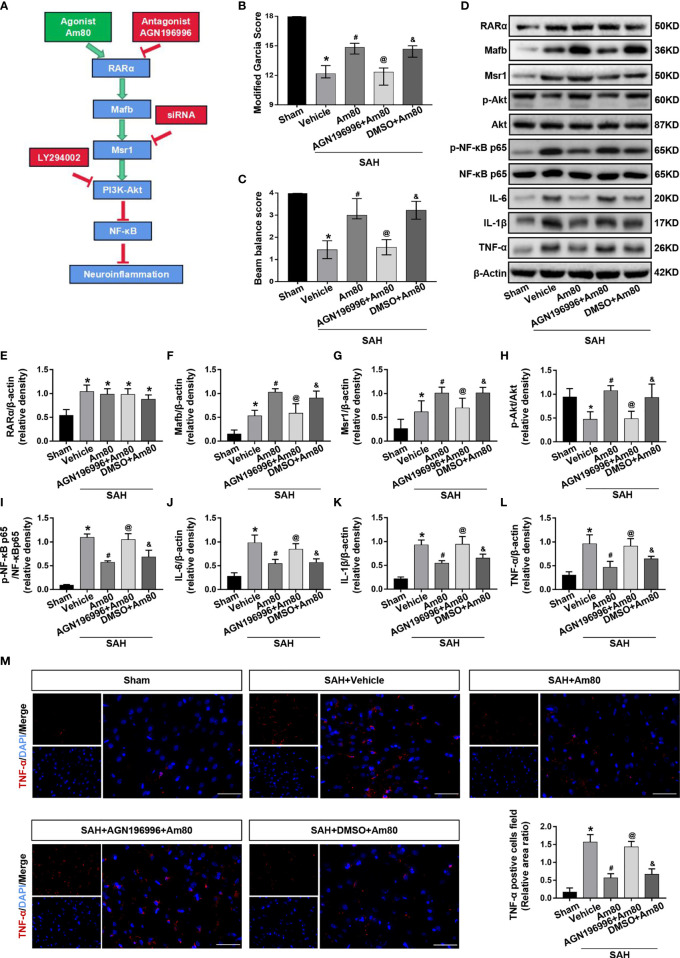
AGN196996 abolished the protective effects of Am80. **(A)** Schematic diagram of experimental design. **(B, C)** The neurological score were evaluated at 24 h after SAH. n = 6 per group. **(D–L)** Western blotting and quantitative analyses of the expression of RARα, Mafb, Msr1, p-Akt, p-NF-kB, *IL-6*, *IL-1β* and *TNF-α*. n = 6 per group. **(M)** Immunofluorescence staining and quantitative analyses of *TNF-α*-positive cells field ratio. Scale bar=50 μm. n = 3 per group. *P < 0.05 *vs* sham group; ^#^ *P* < 0.05 *vs* SAH+vehicle group; ^@^ *P* < 0.05 *vs* SAH+Am80 group; ^&^ *P* < 0.05 *vs* SAH+AGN196996+Am80 group.

To confirm the role of RARα in the protective effects against neuroinflammation, the specific antagonist AGN196996 was used to inhibit the RARα (**Figure 7A**). After administration of the AGN196996, the neurological score of rats was significantly reduced (*P* < 0.05, **Figures 7B, C**). Compared with the Sham group, the expression of Mafb, Msr1, p-NF-κB p65 were significantly increased after SAH; there was no change in level of the RARα (*P* < 0.05, **Figures 7D–I**). Conversely, p-Akt level was reduced in the SAH+vehicle groups (*P* < 0.05, **Figures 7D–I**). The expression of Mafb, Msr1 and p-Akt increased significantly after Am80 treatment, but the expression of p-NF-κB p65, *IL-6*, *IL-1β* and *TNF-α* decreased significantly under the same conditions. (*P* < 0.05, **Figures 7D–I**).Immunofluorescence revealed that the area of *TNF-α*-positive cells in brain cortex after SAH was significantly increased when compared with the Sham group (*P* < 0.05, **Figure 7M**). Am80 treatment reduced the fluorescence area of *TNF-α*-positive cells (*P* < 0.05, **Figure 7M**). Conversely, the protective effects of Am80 were reversed by AGN196996 (*P* < 0.05, **Figure 7M**). The mRNA expression of *IL-6*, *IL-1β*, and *TNF-α* were consistent with the protein result (*P* < 0.05, **Supplementary Figures 1B–D**).

### Am80 Activated RARα to Attenuate Neuroinflammation Through Mafb/Msr1/PI3k-Akt/NF-κB Pathway

**Figure 8:**
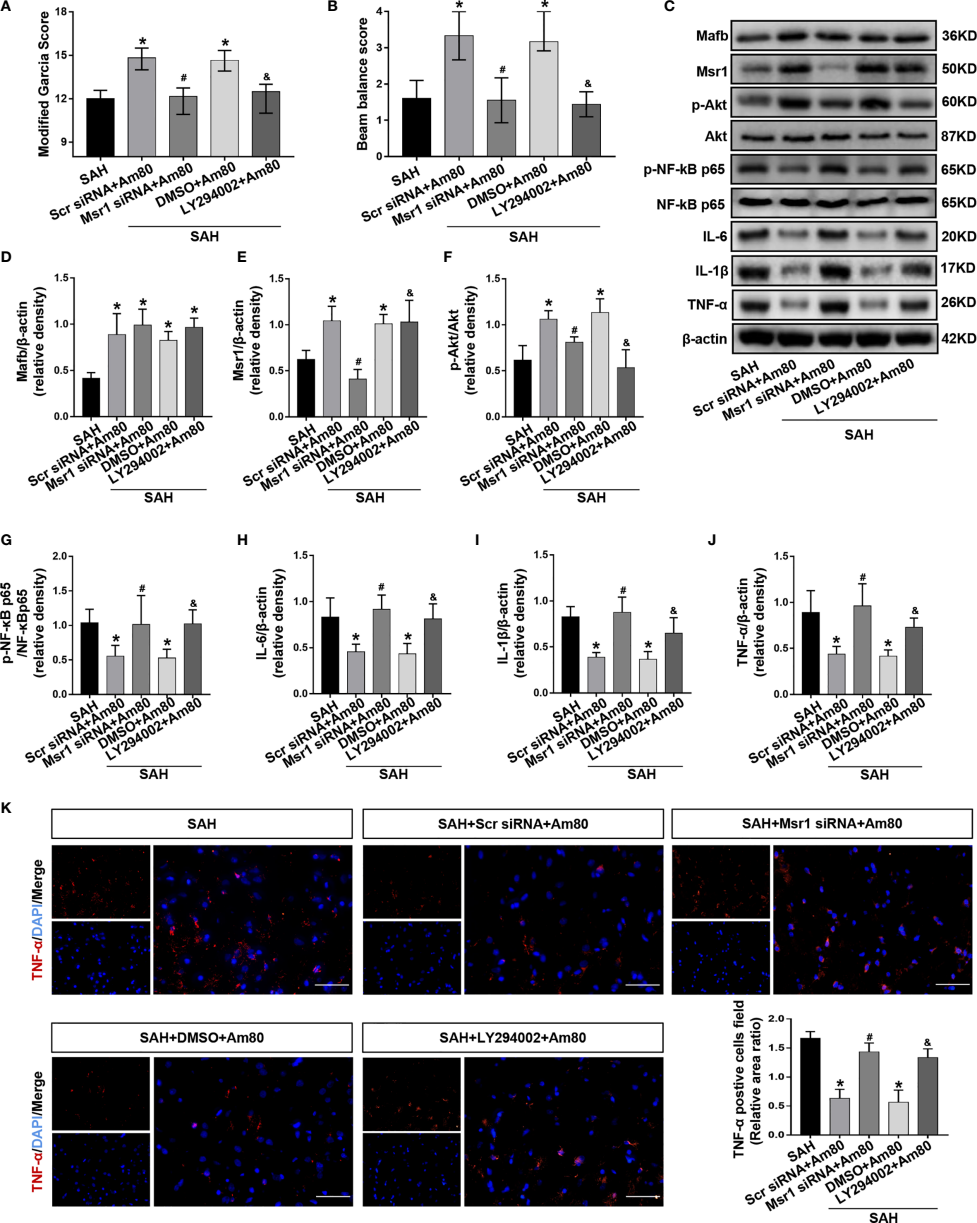
Msr1 siRNA and LY294002 abolished the anti-neuroinflammation effects of Am80. **(A, B)** The neurological score were evaluated at 24 h after SAH. n = 6 per group. **(C–J)** Western blotting and quantitative analyses of the expression of Mafb, Msr1, p-Akt, p-NF-kB, *IL-6*, *IL-1β* and *TNF-*α. n = 6 per group. **(K)** Immunofluorescence staining and quantitative analyses of *TNF-α*-positive cells field ratio. Scale bar = 50 μm. n=3 per group. *P < 0.05 *vs* SAH group; ^#^ *P* < 0.05 *vs* SAH+Scr siRNA+Am 80 group; ^&^ *P* < 0.05 *vs* SAH+DMSO+Am 80 group.

The rat neurological function scores significantly decreased after administration of Msr1 siRNA and LY294002 when compared with the SAH+Am80 group (*P* < 0.05, **Figures 8A, B**).

In the SAH+Msr1 siRNA+Am80 group, the Msr1siRNA abolished the anti-inflammation effects of Am80, thus leading to a reduction in the expression of Msr1 and p-Akt. Meanwhile, the expression of p-NF-κB-p65 and inflammatory cytokines were upregulation when compared with the SAH+Am80 +Msr1 scrRNA group (*P* < 0.05, **Figures 8C–J**). Compared with the SAH+DMSO+Am80 group, pretreatment with LY294002 downregulated the expression of p-Akt but increased the expression of p-NF-κB-p65, *IL-6*, *IL-1β*, and *TNF-α* (*P* < 0.05, **Figures 8C–J**). Immunofluorescence staining further revealed that the fluorescence area of *TNF-α*-positive cells in the SAH+Am80 group was significantly decreased when compared with the SAH+Vehicle group (*P* < 0.05, **Figure 8K**). The administration of Msr1 siRNA and LY294002 reversed these changes (*P* < 0.05, **Figure 8K**). Furthermore, the mRNA expression of *IL-6*, *IL-1β* and *TNF-α* were decreased by treatment with Am80, whereas the effects were abolished by Msr1 siRNA and LY294002. (*P* < 0.05, **Supplementary Figures 1E–G**).

### Activation of RARα Attenuated Neuroinflammation by Inhibiting Nuclear Transcription of NF-κB *In Vitro* Model of SAH

**Figure 9:**
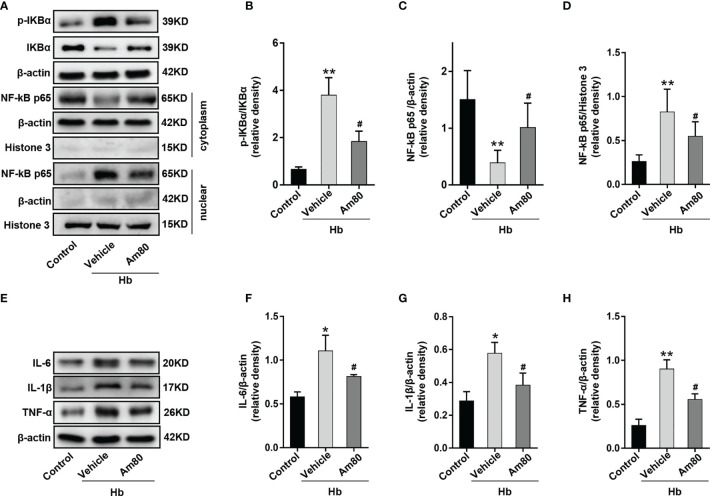
Am80 administration suppressed the nuclear transcription of NF-κB p65 *in vitro* model of SAH. **(A–D)** Western blotting and quantitative analyses of the expression of p-IKB-α and NF-kB p65 in microglia after co-cultivation. **(E–H)** Western blotting and quantitative analysis of the expression of *IL-6*, *IL-1β* and *TNF-α* in microglia after co-cultivation. n = 3 per group. *P < 0.05 *vs* control group; **P < 0.01 *vs* control group; ^#^ *P* < 0.05 *vs* Hb+vehicle group.

To investigate whether the mechanism of Am80 inhibiting inflammation involves nuclear transcription of NF-κB. Western blotting results showed that compared with the control group, the expression of phosphorylated IKBα in BV2 cells increased after Hb induction, which was consistent with the nuclear transcription trend of NF-κB-p65 (*P* < 0.01, **Figures 9A–D**). Compared with the Hb+Vehicle group, the expression of p65 in the cytoplasm increased and the expression of p65 in the nucleus decreased after Am80 administration, which means that the nuclear transcription of p65 was inhibited. Meanwhile, the phosphorylation of IκBα was significantly inhibited (*P* < 0.05, **Figures 9A–D**). Western blot results of inflammatory cytokines showed that the expression of *IL-6*, *IL-1β* and *TNF-α* increased significantly after Hb induction when compared with the Control group (*P <*0.05, **Figures 9E–H**). In contrast, Am80 treatment significantly inhibited the expression of inflammatory cytokines after Hb induction (*P* < 0.05, **Figures 9E–H**).

## Discussion

The current study offers several novel findings with regard to the molecular mechanisms underlying EBI after SAH. First, the expression of RARα, Mafb and Msr1 were co-localized in the microglia and increased after SAH. Secondly, Am80- activated RARα significantly attenuated neurological deficits, the permeability of the BBB, and ameliorated brain edema following SAH. Third, activation of the RARα significantly reduced neuronal injury both *in vivo* and in an *in vitro* model of SAH. Fourth, Am80 treatment inhibited the excessive activation of microglia and promoted M1-to-M2 phenotypic polarization. Fifth, the inhibition of RARα with AGN196996 reversed the protective effects of Am80; this enhanced the expression of downstream inflammatory cytokines (*IL-6*, *IL-1β*, and *TNF-α*). Sixth, Msr1 siRNA and LY294002 abolished the anti-neuroinflammatory effects of Am80 in a manner as the inhibition of p-Akt expression and enhanced the expression levels of p-NF-kB p65; these effects were related to the downregulation of Msr1, p-Akt, and the upregulation of p-NF-κB-p65. In addition, we found that the activation of RARα suppressed the nuclear translocation of NF-κB p65 after SAH. Based on these results, we postulate that the activation of RARα attenuated neuroinflammation by promoting M1-to-M2 phenotypic polarization of the microglia and by regulating the Mafb/Msr1/PI3K-Akt/NF-κB pathway.

Morphological changes in the microglia are known to reflect their activation state (26). When injury occurs, activated microglia develop enlarged cell bodies, shortened processes, and their cell morphology becomes amoeba-like; they also adopt a phagocytic function (26, 32). As the immune cells of the CNS, the morphological changes of microglia are closely related to the functions (33, 34). We examined four key morphological parameters to evaluate changes in microglial morphology following SAH, including fractal dimension, density, area, and perimeter. Results showed that microglial morphology improved significantly following Am80 treatment. The function of microglia not only mediates the endogenous immune response of CNS injury through the release of cytokines and chemokines, but also plays a positive role in the resolution and recovery of inflammation (32). These distinct roles may be attributed to the different microglia phenotypes. M1 microglia release inflammatory cytokines such as *IL-1β*, *TNF-α* and *IL-6* to promote inflammation and aggravate brain damage. In contrast, M2 microglia release anti-inflammatory mediators, including *IL-4*, *IL-10*, and *TGF-β*, to produce beneficial neuroprotective effects (32). It should be noted that M1/M2 typing was originally classified according to the different activation pathways of macrophages (35). However, activated microglia and macrophages can express the same M1 and M2 markers, which makes it difficult to use markers to distinguish and assess the main immune cells that cause neuroinflammation (36, 37). Therefore, the combined term macrophage/microglia is still widely used (38). Recent studies have found that the early neuroinflammatory response after SAH originates from resident microglia rather than infiltrating macrophages (39). Within the EBI after SAH, microglia responded immediately, whereas peripheral infiltrating macrophages were found in the brain parenchyma after 72 hours (40, 41). Zheng’s study also confirmed that although the disruption of the blood-brain barrier allows peripheral macrophages to enter the CNS after SAH, Iba-1-positive cells are more originated from resident microglia (42). More interestingly, microglia expressing both M1 and M2 phenotypes can be observed after SAH, which is considered to be an intermediate state of microglial transformation. Depending on the intervening conditions, bipolar microglia can differentiate into two distinct phenotypes (42, 43). At the same time, an increase in M2 phenotype microglia after SAH, which is associated with better neurological prognosis, has also been demonstrated in many studies (7, 44, 45). These results suggest that promoting the transition of microglia from M1 to M2 phenotype in the early stage after SAH may be beneficial to reduce neuroinflammatory damage after SAH and accelerate the recovery of neurological function. In the present study, activation of the RARα promoted M1-to-M2 phenotypic polarization of microglia in an *in vitro* model of SAH. Apoptosis, as an important regulatory mechanism of cell death, is involved in the pathological processes underlying many diseases (46, 47). Activation of the RARα with Am80 significantly suppressed neuronal apoptosis after SAH; this was consistent with previous research (14) and suggests that the activation of RARα attenuates the neurotoxic effects of activated microglia by suppressing the production of inflammatory cytokines.

We identified RARα as the primary candidate for regulating the effects of microglia after SAH. It is reported that activation of the RARα reduces lung inflammation in mice by inhibiting the transcription of inflammatory cytokines (48). In kidney disease, the activation of RARα confers renal protection by inducing podocyte differentiation and by inhibiting proliferation and inflammation (49). RARα also represents a promising potential therapeutic target in neurodegenerative diseases. Treatment with RARα agonists has been proven to reduce congenital neuroinflammation, improve phagocytosis, and reduce neuropathology in animal models of AD (50–52). As an agonist of RARα, Am80 has already been approved for the treatment of acute promyelocytic leukemia (53). Recently, studies have reported that Am80 represents an effective treatment for treating diseases of the central nervous system, such as ischemic stroke and intracerebral hemorrhage (14, 21). However, previous research has not investigated Am80 as a treatment for SAH, and the specific protective mechanisms after RARα activation still remain unclear. In this study, our results indicated that administration of Am80 significantly improved neurological outcomes after SAH by suppressing the excessive activation of microglia and downregulating the release of inflammatory cytokines. Meanwhile, the results *in vitro* experiments confirmed that Am80 can promote the phenotypic transformation of microglia from M1-Pro-inflammatory to M2-Anti-inflammatory and reduce the injury of neuronal apoptosis. Collectively, these results indicate that Am80 can improve neurological outcomes after SAH. In addition, Am80 can easily penetrate the blood-brain barrier; this means that it can be administered intravenously or orally, and is suitable for clinical applications (54).

As a member of the scavenger receptor family, In the CNS, Msr1 is mainly expressed by microglia and plays a role in removing amyloid, senescent cell debris and various cell damage molecules (55). In previous studies, the expression of Msr1 was enhanced by Mafb and eliminates DAMPs following ischemic stroke *via* endocytosis to reduce the release of inflammatory cytokines by exogenous ligands (21). In addition, Msr1 can promote macrophage M2-like polarization by activating the PI3K-Akt pathway in osteogenic differentiation after fracture (56). In this study, we confirmed that Msr1-mediated enhancement of Akt phosphorylation and inhibition of NF-κB phosphorylation can be abolished by PI3K inhibitors. These results indicate that the PI3K-Akt/NF-κB pathway mediated by Msr1 is involved in the regulation of neuroinflammation after SAH.

NF-κB is an important nuclear transcription factor in cells and participate in the response of cells to stimuli, such as stress, cytokines, free radicals, and bacterial or viral antigens. Misregulation of NF-kB involves many diseases, such as hepatitis, pancreatitis, and brain diseases (57, 58). When the NF-κB signaling pathway is activated, the p65 subunit of the NF-κB complex translocates into the nucleus, binds to DNA, and initiates the transcription of inflammatory cytokines (30). Therefore, inhibiting the NF-kB signal transduction pathway after SAH may be an effective treatment. Our research indicated that Am80 reduces IκBα phosphorylation and p65 nuclear translocation. In addition, the expression of *IL-1β*, *TNF-α* and *IL-6* also inhibited by Am80, as reported previously (59).

Our results revealed that activation of RARα can improve the neurological outcomes after SAH by reducing inflammation. However, our research has some limitations that need to be considered. First, we did not evaluate the role of Msr1 endocytosis with regards to the reduction of neuroinflammation after SAH. Second, the *in vitro* induction of oxyhemoglobin does not completely mimic the process of SAH *in vivo*. Finally, although we have demonstrated that RARα activation confers protective effects on neuronal injury *via* the Mafb/Msr1/PI3K-Akt/NF-κB axis, at least in part, the protective effects of RARα in other pathways has not been excluded; these potential mechanisms need to be investigated further.

## Conclusion

In this study, Our results revealed that activation of RARα with Am80 improved neurological outcome and attenuated neuroinflammation of EBI after SAH by promoting M1-to-M2 phenotypic polarization of microglia and by regulating the Mafb/Msr1/PI3K-Akt/NF-κB pathway. Therefore, RARα may be a potential therapeutic target for reducing brain injury after SAH.

## Data Availability Statement

The original contributions presented in the study are included in the article/**Supplementary Material**. Further inquiries can be directed to the corresponding author.

## Ethics Statement

The animal experiments involved in this study have been reviewed and approved by the Animal Experiment Center of the First Affiliated Hospital of Harbin Medical University (NO:2020047).

## Author Contributions

YT and HS: designed and conceived this research. YT and YL: animal experiments. BL and YZ: cell experiments. JS and PW: data analysis. YT and GC: prepared the manuscript. YT, HS, YL, BL, and CX: revised the manuscript. YT, BL, and YL contributed equally. All authors contributed to manuscript revision, read, and approved the submitted version.

## Funding

This study was supported by the National Natural Science Foundation, China (82071309, 82101383 and 81901190) and Heilongjiang Postdoctoral Foundation, China (LBH-Z20169) and China Postdoctoral Science Foundation (2021MD703829).

## Conflict of Interest

The authors declare that the research was conducted in the absence of any commercial or financial relationships that could be construed as a potential conflict of interest.

## Publisher’s Note

All claims expressed in this article are solely those of the authors and do not necessarily represent those of their affiliated organizations, or those of the publisher, the editors and the reviewers. Any product that may be evaluated in this article, or claim that may be made by its manufacturer, is not guaranteed or endorsed by the publisher.
