# Supplementary material for: Activation of RARα Receptor Attenuates Neuroinflammation After SAH via Promoting M1-to-M2 Phenotypic Polarization of Microglia and Regulating Mafb/Msr1/PI3K-Akt/NF-κB Pathway
Source: Front Immunol. 2022 Feb 14;13:839796. doi: 10.3389/fimmu.2022.839796 (PMC8882645; doi:10.3389/fimmu.2022.839796)
Supplement: Supplementary file 1 [file DataSheet_1.docx]

**SUPPLEMENTARY FIGURES:**


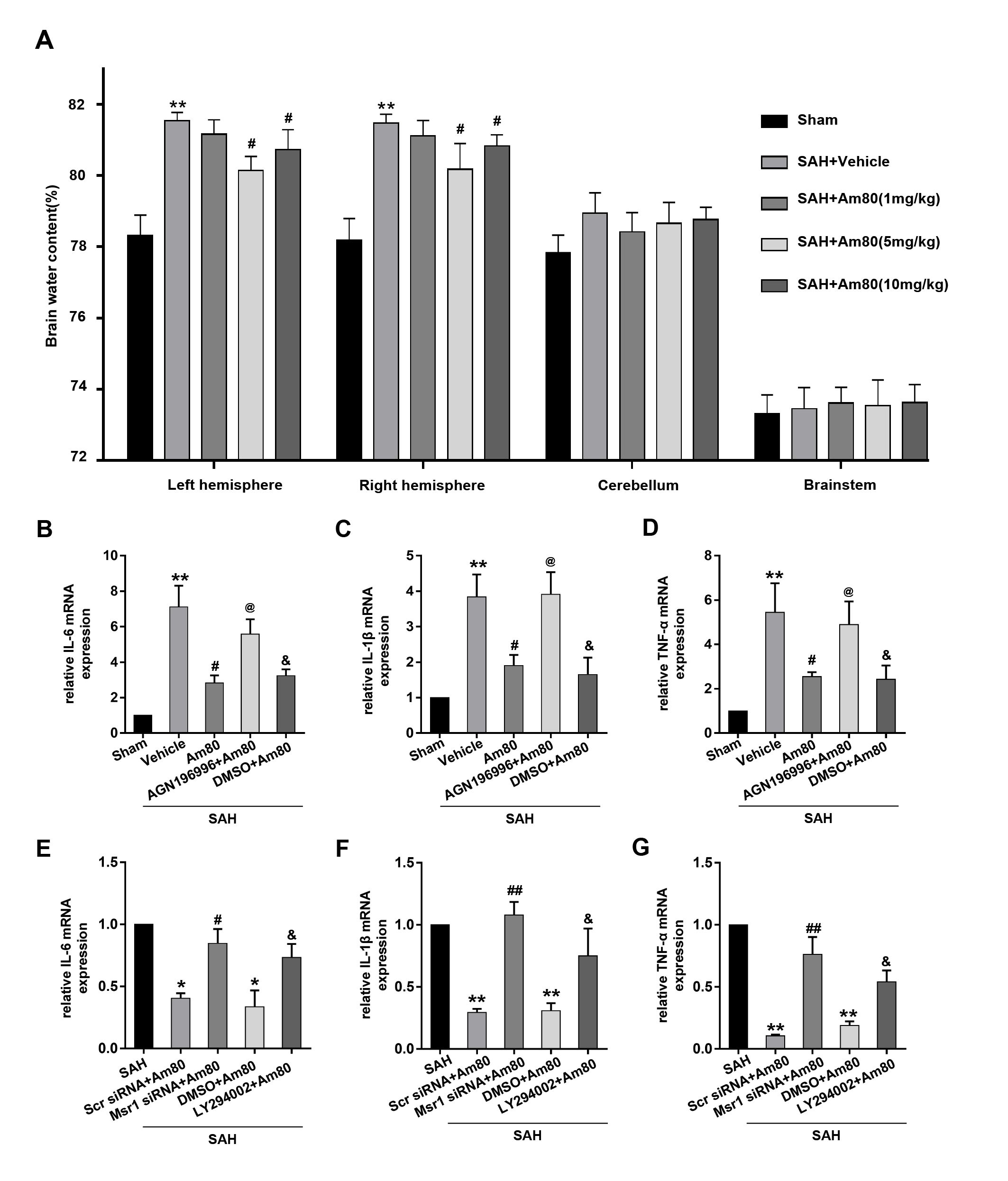


**Supplementary Figure 1.**(**A**) Am80 attenuated brain water content at 24 h after SAH. Quantification of brain water content in the left hemisphere, right hemisphere, cerebellum, and brainstem at 24 h after SAH. n=6 per group. Error bars represent mean ± SD. ******P*<0.05 vs sham group; *******P*<0.01 vs sham group; **^#^***P*<0.05 vs SAH+vehicle group. (**B**)-(**D**) Am80 suppressed the mRNA expression of inflammatory factors (*IL-6*, *IL-1β* and *TNF-α*) at 24 h after SAH; these changes were abolished by AGN196996. Quantitative analyses of the mRNA expression of IL-6, *IL-1β* and *TNF-α*. n=3 per group. ******P*<0.05 vs sham group; *******P*<0.01 vs sham group; **^#^***P*<0.05 vs SAH+Am80 group; **^@^***P*<0.05 vs SAH+Am80 group; **^&^***P*<0.05 vs SAH+AGN196996+Am80 group. (**E**)-(**G**) Am80 suppressed the mRNA expression of inflammatory factors (*IL-6*, *IL-1β* and *TNF-α*) at 24 h after SAH; these changes were abolished by Msr1 siRNA and the PI3k selective inhibitor LY294002. Quantitative analyses of the mRNA expression of *IL-6*, *IL-1β* and *TNF-α*. n=3 per group. ******P*<0.05 vs SAH group; *******P*<0.01 vs SAH group; **^#^***P*<0.05 vs SAH+Scr siRNA+Am80 group; **^##^***P*<0.01 vs SAH+Scr siRNA+Am80 group; **^&^***P*<0.05 vs SAH+DMSO+Am80 group.


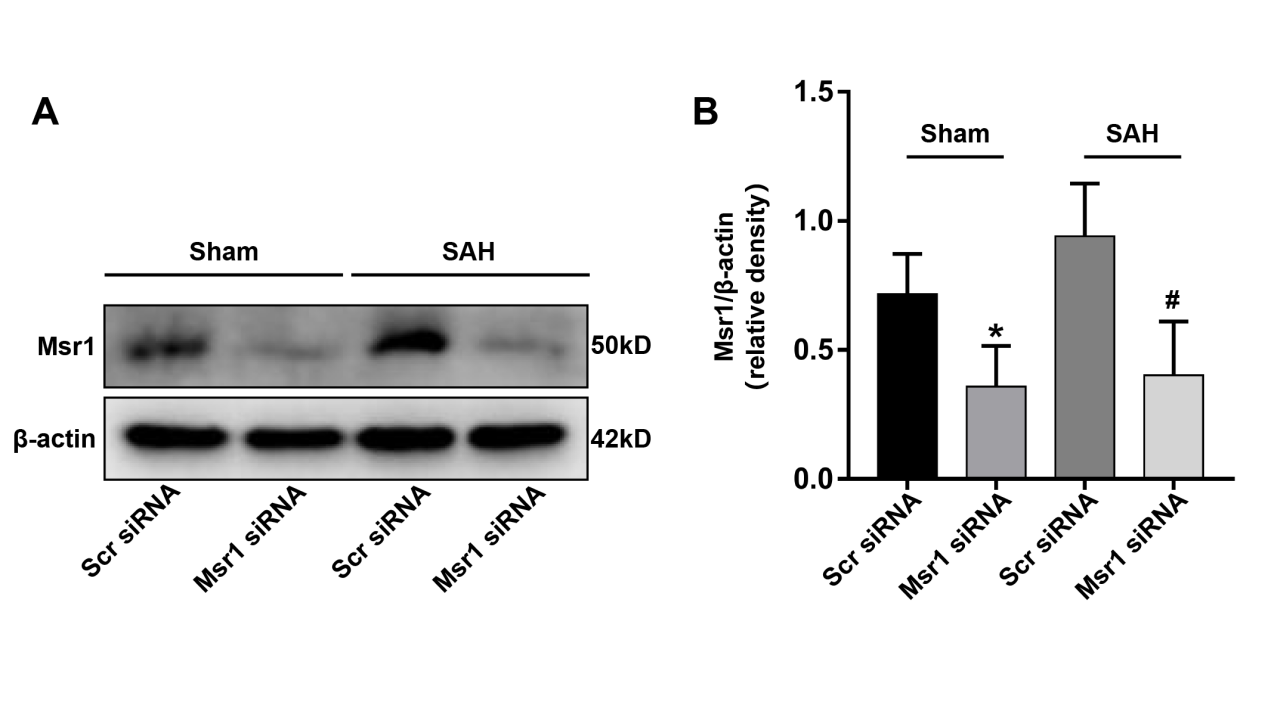


**Supplementary Figure 2.**

(**A**)-(**B**) Representative western blot bands and quantification of Msr1 demonstrated the knockdown efficacy of Msr1 siRNA in Sham and SAH groups. n=3 per group. ******P*<0.05 vs Sham+Scr siRNA group; **^#^***P*<0.05 vs SAH+Scr siRNA group.

**SUPPLEMENTARY TABLE 1**

| Primers used in real-time qRT-PCR reactions | | |
| --- | --- | --- |
| Gene | Upstream primers | Downstream primers |
| *IL-6* | 5'-TAGTCCTTCCTACCCCAACTTCC-3' | 5'-TTGGTCCTTAGCCACTCCTTC-3' |
| *IL-1β* | 5'-CCTATGTCTTGCCCGTGGAG-3' | 5'-CACACACTAGCAGGTCGTCA-3' |
| *TNF-α* | 5'-GCCACCACGCTCTTCTG-3' | 5'-GCAGCCTTGTCCCTTGA-3' |
| GAPDH | 5'-CCCACTAACATCAAATGGGG-3' | 5'-ATCCACAGTCTTCTGGGTGG-3' |
